# Appendiceal Intraluminal Gas: A CT Marker for Gangrenous Appendicitis

**DOI:** 10.1155/2021/7191348

**Published:** 2021-11-22

**Authors:** Chantelle Ip, Edward H. Wang, Michael Croft, Wanyin Lim

**Affiliations:** ^1^Royal Adelaide Hospital, Port Road, Adelaide 5000, Australia; ^2^Flinders Medical Centre, Flinders Dr, Bedford Park 5042, Australia; ^3^Dr Jones and Partners, 226 Greenhill Road, Eastwood 5065, Australia

## Abstract

**Introduction:**

This manuscript aims to investigate the amount of intraluminal gas in acute, nonperforated appendicitis identified on computed tomography (CT) in diagnosing gangrenous appendicitis.

**Methods:**

This is a retrospective observational, case-control study with consecutive data collected at a tertiary institution over a two-year period, of patients with CT-diagnosed acute appendicitis who subsequently went on for surgery within 48 hours. Patients who were less than 16 years old, who had an interval between CT and surgery of more than 48 hours, or with CT evidence of appendiceal perforation were excluded. Images were independently assessed by 3 radiologists for intraluminal gas, and the results were then correlated with reference standards obtained from surgical and histopathology reports for the diagnosis of nongangrenous versus gangrenous appendicitis. The sensitivity, specificity, and predictive values of CT intraluminal gas in gangrenous appendicitis were calculated.

**Results:**

Our study identified 93 patients with nonperforated acute appendicitis who underwent surgery within the stated timeframe. Intraluminal gas in the appendix was identified in 26 patients (28%), of which 54% had macroscopic and/or microscopic evidence of gangrenous appendicitis. This is in contrast to the subgroup of patients who did not have intraluminal gas (72%), of which only 33% had gangrenous appendicitis. The specificity of intraluminal gas for gangrenous appendicitis is 79%, with a negative predictive value of 86% and likelihood ratio of 1.85.

**Conclusion:**

In cases of established acute appendicitis, the presence of intraluminal gas is a moderately specific sign for gangrenous complication. This is worth reporting as it can help prognosticate and triage patients accordingly, for a timelier surgical management and a better outcome.

## 1. Introduction

Acute appendicitis is an important cause of acute abdominal pain, with appendectomies being the most common emergency procedure performed in Australia [1]. Whilst acute appendicitis is a clinical diagnosis, CT scanning has become an important imaging tool to reduce the rate of negative or unnecessary surgeries by identifying other causes of abdominal pain which can mimic appendicitis. Appendicitis can have different clinical presentations, with overlapping clinical features between acute uncomplicated and complicated appendicitis such as perforation and abscess collection [2], and hence, imaging also plays an important role in differentiating between the simple and complicated cases [3]. These have important implications for patient management pathways, prognosis, and length of hospital stay.

There have been many studies focusing on CT signs differentiating perforated from nonperforated cases of appendicitis; however, few studies have focused on signs that differentiate simple phlegmonous appendicitis from gangrenous appendicitis in the nonperforated appendix [4]. This distinction is important as these two classifications of nonperforated appendicitis are associated with very different prognoses, with time-sensitive treatment pathways. Gangrenous appendicitis is estimated to occur in 17% of cases of acute appendicitis [5] and is associated with increased rates of complications such as intra-abdominal abscesses and appendiceal perforation [1] if surgical management is delayed.

The presence of intraluminal air within the appendix was traditionally thought to be a useful sign to exclude appendicitis, indicating that the appendix was patent, nonobstructed, and not inflamed [6]. However, there is increasing evidence that intraluminal gas on CT is common in appendicitis and may be seen in 19–27% of cases [7, 8]. One hypothesis is that there is an absence of intraluminal air in an acutely inflamed appendix due to gas displacement by intraluminal fluid and increased pressure. However, as the inflammation progresses towards gangrenous complication, intraluminal air develops in a similar pathophysiology as in the development of pneumatosis intestinalis.

This study aims to assess the presence of intraluminal air in the appendix on CT, an indicator for the gangrenous appendix in cases of acute nonperforated appendicitis.

## 2. Materials and Methods

*Statement of Ethics*. Ethical approval was granted for this project by the Institutional Human Research Ethics Committee (R20190408).

*Data Collection*. Data were collected from the picture archiving and communication system (PACS) of a tertiary hospital in South Australia using the keyword search “appendix, appendiceal inflammation, appendicitis.” Consecutive patients over a two-year period (2018 and 2019) with CT prior to appendectomy for acute appendicitis were included in the study. Data extracted from existing databases included patient age, sex, time of CT, abdomen CT images, time of surgical intervention, operation report, and surgical histopathology report.

*Exclusion Criteria*. Exclusion criteria included the following patients with Ct-diagnosed appendicitis who did not proceed with surgery within 48 hours of CT scan, patients younger than 18 years of age, and patients with appendiceal perforation evident on CT at time of presentation.

Patients younger than 18 were excluded due to the small number of pediatric cases presenting to this adult institution and the increasing evidence that CT is not the recommended imaging modality in this cohort [4, 9].

*Retrospective Image Review*. The CT images, comprising volumetric CT scans with multiplanar reconstruction at 1 and 3 mm slices, were independently reviewed by three radiologists, including two registrars and one consultant radiologist with 4 years of experience, for the presence of appendiceal gas and perforation. Other signs of inflammation were noted to confirm diagnosis of appendicitis, and these included increased appendiceal diameter, wall thickness and enhancement, and associated appendicolith and periappendiceal fat stranding or collection [11].

Cases with CT-evident perforated appendicitis were then excluded. The CT findings indicative of perforation include a focal defect in the enhancing appendiceal wall, surrounding phlegmon or abscess, extraluminal air, and extraluminal appendicolith [10].

The reviewers were provided patient age and sex but were blinded to the original CT report and the subsequent surgical and histopathology reports. Any disagreements between the three reviewers were resolved by reviewing and discussing the images and correlating with the original CT report.

*Reference Standards*. Reference standards included the existing surgical records and histopathology reports. A definitive diagnosis of appendicitis was determined by the histopathologic documentation of transmural inflammation, ulceration, or thrombosis [12]. Gangrenous appendicitis was defined macroscopically as either a friable appendix with green, purple, or black colour changes or microscopically as the presence of transmural inflammation with necrosis. The presence of appendiceal perforation was determined by surgical documentation of visible perforation that was not caused by surgical handling or histopathologic documentation of appendiceal wall defect due to transmural necrosis.

*Statistical Analysis*. A readily available statistics calculator was used to calculate the sensitivity, specificity, positive and negative likelihood ratios, and positive and negative predictive values of CT intraluminal gas in nonperforated gangrenous appendicitis.

For sample size estimation, as gangrenous appendicitis is estimated to occur in 17% of cases of acute appendicitis [5], we had a minimum target of 100 total studies. This would result in an estimated 17 cases of gangrenous appendicitis and 83 cases of nongangrenous appendicitis. This sample size would be adequate to demonstrate a medium-to-large effect, with an estimated power of 0.8 and an alpha value of 0.05. An effect of this scale would be sufficient for our hypothesis.

## 3. Results

*Demographic Information*. The PACS database search yielded 305 patients. Of these, 135 patients had CT-diagnosed appendicitis prior to appendectomy within 48 hours of the CT scans. Of these, 3 patients younger than 18 years of age were excluded. 6 patients who underwent negative appendicectomies (four cases of histopathologically noninflammed appendixes, one case of leukaemia with appendiceal involvement, and one case of appendiceal adenocarcinoma) were excluded. Finally, there were 31 cases of CT-evident perforated appendicitis which were also excluded.

The final study group comprised 93 patients with a mean (±SD) age of 49.0 ± 14.7 years (range, 19–95 years). There were 38 women (mean age, 52.6 ± 16.9 years; range, 20–95 years) and 55 men (mean age, 46.6 ± 12.3 years; range, 19–71 years).

*Retrospective Review*. There were 57 cases of surgically or pathologically confirmed simple (nongangrenous) acute appendicitis and 36 cases of nonperforated gangrenous appendicitis. The prevalence of gangrenous appendicitis in our study group was 39% (26/93).

Appendiceal intraluminal air on CT was present in 26 patients (26/93; 28%) and absent in 67 (67/93; 72%).

Of the 26 with appendiceal intraluminal air detected on CT, 14 patients (14/26; 54%) had histological/surgical evidence of gangrenous appendicitis and 12 patients (12/26; 46%) did not. Of the 67 without CT findings of appendiceal intraluminal air, 22 patients (22/67; 33%) had evidence of gangrenous appendicitis intraoperatively or surgically and 45 patients (45/67; 67%) did not (Table 1).

**Figure 1:**
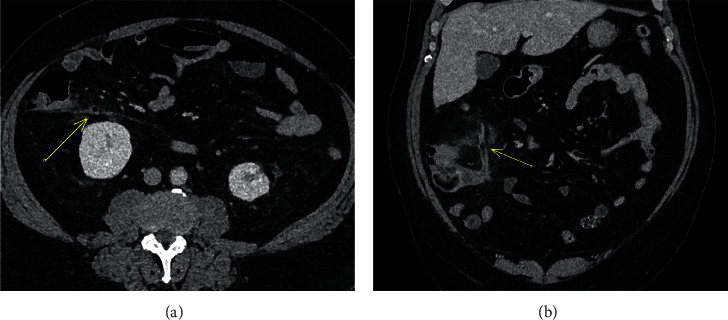
A 64-year-old man with axial (a) and coronal (b) CT abdomen in the portal venous phase showing periappendiceal fat stranding and appendiceal wall hyperenhancement, confirming the clinical suspicion of acute appendicitis. There is a focus of intraluminal gas in the body of the appendix (arrow). Gangrenous appendicitis was confirmed intraoperatively and on subsequent histopathology.

**Figure 2:**
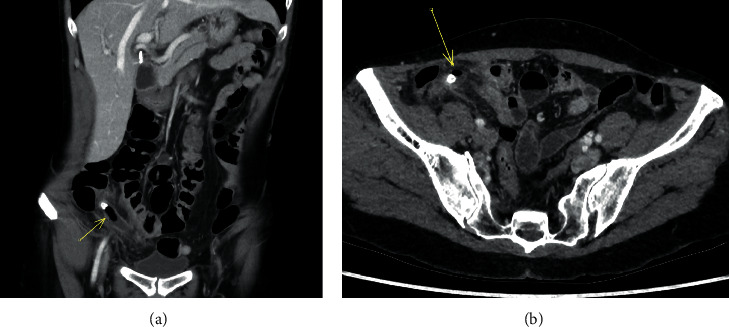
A 58-year-old woman with clinical suspicion of acute appendicitis. Coronal (a) and axial (b) CT abdomen in the portal venous phase shows a distended appendix with periappendiceal fat stranding. There is intraluminal gas adjacent to the appendicolith towards the tip. Gangrenous appendicitis was confirmed on subsequent histopathology report and intraoperatively.

**Figure 3:**
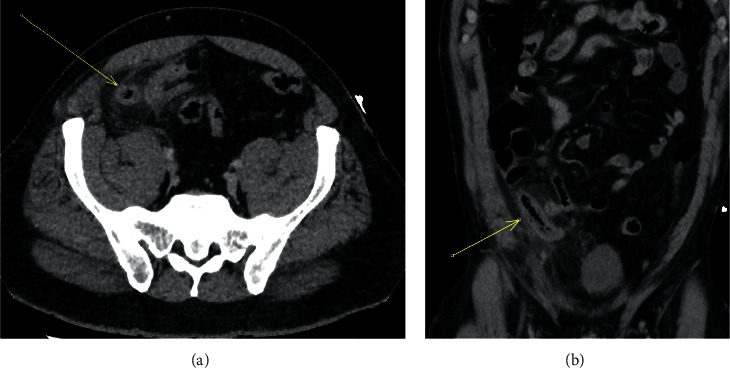
A 45-year-old man with appendiceal intraluminal gas (arrow) on noncontrast CT in axial (a) and coronal (b) projections. Histological and surgical evidence of gangrenous appendicitis. Note the distended appendix with a thickened wall and surrounding fat stranding.

**Figure 4:**
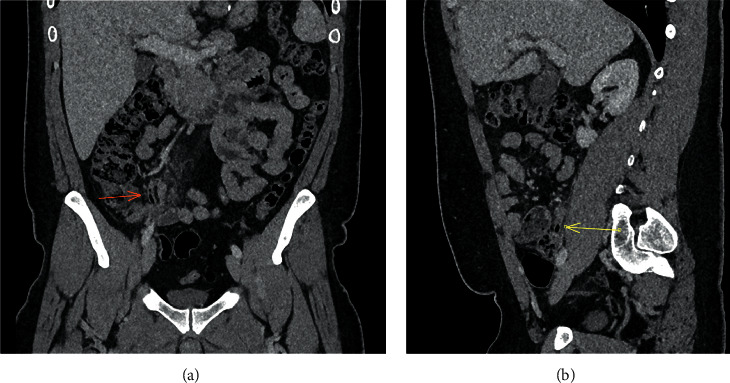
A 33-year-old man with appendiceal intraluminal gas on CT (arrow). Coronal (a) and sagittal (b) CT abdomen in the portal venous phase. The appendix diameter is still within normal limits, but there is low-grade surrounding fat stranding. This is a false-positive case, with histological/surgical reports confirming uncomplicated suppurative acute appendicitis, without gangrenous complication.

Figures 1–3 exhibit cases of CT-evident acute appendicitis with the presence of appendiceal intraluminal gas; gangrenous appendicitis was confirmed intraoperatively within 24 hours for all three patients and then histologically.  Figure 4 exhibits a false-positive case; the appendix has low-grade surrounding fat stranding with appendiceal intraluminal gas; uncomplicated suppurative appendicitis was confirmed within 24 hours both surgically and histologically.

## 4. Statistical Analysis

As a marker for the gangrenous appendix in nonperforated appendicitis, intraluminal air on CT was found to have a specificity of 79% (95% CI, 66–89%), positive likelihood ratio of 1.85 (95% CI, 1.0–3.5), and negative predictive value of 86% (95% CI, 82–89%) (Table 2). The accuracy of correctly classifying the patient's appendicitis based on CT findings of intraluminal gas is 72%.

## 5. Discussion

Gangrenous appendicitis is associated with higher rates of complications such as perforation, abscess, and faeculent peritonitis. These have significant implications in patients, as they are associated with increased length of hospital stay, higher morbidity and mortality, and higher rates of open incision surgery and hospital readmission [1].

Due to the wide variation of clinical findings on examination in cases of acute appendicitis, CT has been a useful imaging modality to decrease the rate of negative appendicectomies and identify complicated cases such as perforation [13, 14]. There are many studies researching CT markers to differentiate perforated versus nonperforated appendicitis. However, when there is no CT evidence of perforation, there is scant data on whether there are CT markers which can differentiate between acute suppurative appendicitis and gangrenous appendicitis. It would be useful if radiologists could make this distinction, as accurately identifying patients with a higher risk for gangrenous complication would warrant more urgent appendicectomies which can, down the line, provide better service for the patients.

It is widely acknowledged that intraluminal gas in the appendix on CT is very commonly seen in the normal appendix [6, 7] as well as in a smaller percentage of patients with acute appendicitis [7, 8]. The diagnosis of acute appendicitis is often first made utilising a combination of other CT signs of appendiceal inflammation such as increased appendiceal diameter (6 mm), wall thickening and altered enhancement, and associated appendicolith and periappendiceal fat stranding or collection. In fact, it is much more common in cases of nonperforated acute appendicitis to have an absence of intraluminal air in the appendix (up to 66%) [7] as appendiceal inflammation from luminal obstruction leads to intraluminal fluid collection and displacement of prominent gas locules. Histologically, this is seen as transmural inflammation with infiltration of neutrophils. Further inflammation with gangrenous transformation, histologically seen as progression to focal necrosis, can then lead to the development of new gas locules from the gas-forming organisms, which can then be evident on CT.

Our study identified 93 consecutive patients with CT prior to appendectomy for nonperforated acute appendicitis between 2018 and 2019. Of these, 67 patients (67/93; 72%) demonstrated no gas in the appendix on CT. This is in keeping with other studies showing that intraluminal gas is more commonly absent in acute appendicitis. From this subgroup, only 33 percent (22/67) had macroscopic and/or microscopic evidence of gangrenous appendicitis. This is relatively less than the subgroup of patients with gangrenous appendicitis with intraluminal gas on CT, 54 percent (14/26).

From our results, intraluminal gas in nonperforated acute appendicitis on CT has a moderately high specificity of 79 percent, with a negative predictive value of 86 percent. This means that presence of gas is suggestive of suppurative appendicitis. As intraluminal gas may be present in either of these pathologies, it is within expectations that this marker has a lower sensitivity (39%) and positive predictive value (27%).

Even so, our data suggest that patients with acute appendicitis with intraluminal gas on CT are 1.85 times more likely to have gangrenous appendicitis and that the overall probability that an appendix is properly classified as gangrenous vs suppurative based on CT findings of intraluminal gas is 72% (62–81%).

Our study has focused on the intraluminal gas as a standalone finding in predicting gangrenous appendicitis. The specificity and likelihood ratio were externally validated and were similar to the recently published meta-analysis by HY Kim et al., using CT features to differentiate between complicated and uncomplicated appendicitis [11]. The group found that whilst being assessed in isolation, the sensitivity for intraluminal gas in complicated appendicitis is poor; however, if pooled with the other features of appendicitis that we know of, such as periappendiceal fat stranding and appendicolith, the presence of intraluminal gas is a useful feature in predicting complicated appendicitis.

The other limitations included the variability in reporting detail within database surgical and histopathological reports contributed to uncertainty when used as a reference standard. Only reports which stated the specific findings of either a friable appendix with green, purple, or black colour changes or the presence of transmural inflammation with necrosis were deemed positive results. This could have contributed to type II error and increased false-negative cases.

There may also be a discrepancy between the CT images and the surgical/histopathology reports due to multifactorial extrinsic delays to surgery. The patient's clinical presentations which could influence management options, were not available to us. Furthermore, CT abdomen scans without contrast were not excluded, and this may have affected reporting accuracy with missed cases of CT perforation.

Ultimately, appendicitis remains a clinical diagnosis, and currently, CT is employed predominantly in atypical presentations to identify cases suspected to have complications or to exclude other differentials. This could be one of the reasons for our study population having a higher prevalence of gangrenous appendicitis (39%) when compared with other studies (17%).

Whilst the sample size is smaller than what we have initially hoped to achieve, it was calculated based on prevalence of 17%, for 17 cases of gangrenous appendicitis. Given the difference in prevalence of gangrenous appendicitis in our cohort, this study is still considered sufficiently powered.

Future studies in this area may include a large prospective multicentre cohort study, with collaboration between radiologists, surgeons, and pathologists to create a pro forma and standardised criteria for reporting appendicitis, and in particular for gangrenous change. This would provide external validation to our hypothesis. Future studies may also investigate gas patterns and exact location of the appendiceal gas and the relationship of this CT marker with the development of complications (e.g., abscess, perforation, and peritonitis) depending on CT to surgery time. This would aim to provide further evidence for utilising this sign to triage patients for urgent appendicectomies.

## 6. Conclusion

This finding of intraluminal air as an indicator of gangrenous appendicitis is not well known amongst the radiology community, and the manuscript hopes to increase the awareness of this CT feature in the setting of acute appendicitis. Our study demonstrates a moderately high level of specificity (79%) but low sensitivity of intraluminal gas as a CT marker for gangrenous appendicitis in the setting of acute, nonperforated appendicitis. If there is intraluminal gas present, the appendix is 1.9 times more likely to be gangrenous, with moderate accuracy.

What is more important is that combined with other CT findings for acute appendicitis such as increased appendiceal diameter, wall enhancement, or adjacent fat stranding, intraluminal air is an important CT tool as it is a good predictor for gangrenous complication, before progression to perforated appendicitis [11]. Therefore, the presence of this sign in patients is an important feature for radiologists to note and convey to our surgical colleagues, to prompt a more timely surgical management and a better patient outcome.

## Figures and Tables

**Table 1:** Cases of appendicitis with or without CT-detected intraluminal gas, correlated with the final histopathological report of absence or presence of gangrenous appendicitis.

Intraluminal gas on CT	Gangrenous appendicitis	Nongangrenous appendicitis
Present	14	12
Absent	22	45

**Table 2:** Statistical analysis of intraluminal gas in predicting gangrenous appendicitis.

Statistics	Value	95% confidence interval
Specificity	78.95%	66.1–88.6%
Sensitivity	38.89%	23.1–56.5%
Negative likelihood ratio	0.77	0.6–1.0
Positive likelihood ratio	1.85	1.0–3.5
Negative predictive value ^*∗*^	86.32%	82.5–89.4%
Positive predictive value ^*∗*^	27.45%	16.5–42.0%

^*∗*^Disease prevalence = 17% [5] (Emil 2012).

## Data Availability

Data are available from the corresponding author on request.
